# New insights into interstitial cystitis/bladder pain syndrome at single‐cell resolution

**DOI:** 10.1002/bco2.70051

**Published:** 2025-08-04

**Authors:** Tadeja Kuret, Mateja Erdani Kreft

**Affiliations:** ^1^ Institute of Cell Biology, Faculty of Medicine University of Ljubljana Ljubljana Slovenia

**Keywords:** bladder pain syndrome, high throughput, immune cells, interstitial cells, interstitial cystitis, Single‐cell landscape, urothelial cells

## Abstract

**Objective:**

Interstitial cystitis/bladder pain syndrome (IC/BPS) is a chronic inflammatory bladder disorder with unknown aetiology and limited treatment options. Single‐cell RNA‐sequencing (scRNA‐seq) has provided unprecedented insights into cellular heterogeneity in IC/BPS. This review summarizes recent scRNA‐seq findings on bladder cell populations, emphasizing urothelial, interstitial and immune cells.

**Methods:**

A comprehensive analysis of published scRNA‐seq studies was conducted to compare bladder cell subtypes in healthy and IC/BPS‐affected bladders. Differences between IC/BPS patients and mouse models, as well as sex‐specific cellular variations, were examined.

**Results:**

IC/BPS bladders exhibit significant urothelial alterations, including a reduction in UPK3A + umbrella cells and an expansion of progenitor‐like cells with impaired regenerative capacity, linked to TLR3‐NR2F6 signalling. Interstitial cells include three fibroblast subtypes (PDGFRA+, RGS5+ and pro‐inflammatory IL6‐producing fibroblasts), which contribute to fibrosis and inflammation. The immune landscape is characterized by a Th1‐biased response, exhausted CD8 + T cells and reduced regulatory T cells, with HPV infection detected in most IC/BPS patients, suggesting a possible viral aetiology. Cell‐to‐cell interactions are compromised, with enhanced macrophage‐endothelial signalling via CXCL8‐ACKR1 and CXCL2/3‐ACKR1 pathways, highlighting potential therapeutic targets. Notably, sex‐based differences reveal stronger immune activation in females and increased urothelial proliferation in males, potentially explaining the higher IC/BPS prevalence in females.

**Conclusions:**

scRNA‐seq has advanced our understanding of IC/BPS by identifying disease‐associated cell types, signalling pathways and intercellular interactions. Future research should integrate multi‐omics approaches and explore non‐invasive urine‐based scRNA‐seq for improved diagnosis and therapy.

**Abbreviations**
IC/BPS: interstitial cystitis/bladder pain syndrome
HIC: Hunner type
NHIC: non‐Hunner type
scRNA‐seq: single cell RNA‐sequencing
CYP: cyclophosphamide

## 1 INTRODUCTION

Interstitial cystitis/bladder pain syndrome (IC/BPS) is a chronic inflammatory disease of the urinary bladder characterized by the absence of bacterial infection or an identifiable pathological cause. To meet the diagnosis of IC/BPS, patients must experience chronic pelvic pain, pressure or discomfort, and at least one of the following symptoms: increased urinary frequency, urgency or nocturia, persisting for a minimum duration of six months.^1^ The estimated incidence of IC/BPS ranges from 52 to 500 per 100.000 females and is reported to be 10 times higher than in males.^2^

Currently, treatment options for IC/BPS patients range from patient education and oral medications to intravesical instillations, including dimethyl sulfoxide (DMSO), botulinum toxin, chondroitin sulphate, triamcinolone, hyaluronic acid and lidocaine. These treatments mainly provide symptom relief, with no proven long‐term efficacy for all IC/BPS patients.^3^ The heterogeneity of the condition, reflected in multiple clinical phenotypes, likely contributes to the limited success of current therapies and clinical trials.^1^, ^4^ So far, only two IC/BPS phenotypes have been identified based on histologic or cystoscopic findings: Hunner‐type (HIC/BPS), characterized by mucosal lesions with abnormal capillary architecture, and non‐Hunner type (NHIC/BPS), which lacks clear bladder pathology and shows minimal histological changes.^5^

Despite this complexity, most prior studies relied on studies of a specific cell population, the expression of a few genes or proteins and transcriptomic findings from bulk RNA sequencing, which averages gene expression across diverse cell types.^18^, ^19^ The introduction of single‐cell RNA‐sequencing (scRNA‐seq) technology in 2009^20^ has overcome these limitations, enabling comprehensive and unbiased transcriptional profiling of thousands of individual cells simultaneously. This allows for in‐depth characterization of cell phenotypes and functions, identification of rare cell subpopulations and deeper insights into cell‐to‐cell variability, intercellular communication and linking external triggers, like viral infection to urothelial and immune cell responses.^21^ Recently, this approach has been applied to IC/BPS research, enhancing our understanding of this disease (Table 1).

**TABLE 1:** **Summary of major findings from scRNA‐seq in IC/BPS patients or animal models**.

Reference	Patients or mouse models (number)	Cell types sequenced (number)	Main findings
Su et al., 2022^43^	IC/BPS patients (n = 2 HIC/BPS; n = 3 NHIC/BPS); bladder cancer patients (n = 2)	Bladder tissue cells (n = 44.490)	HIC, NHIC/BPS: Th1 biased response, lower levels of Tregs, enriched populations of macrophages expressing *IL1𝛽* and *TNFα*, 3 types of fibroblasts: *PDGFRA+, RGS5 +* and *IL6* producing pro‐inflammatory fibroblasts, interactions between macrophages and endothelial cells via *CXCL8‐ACKR1/CXCL2/3‐ACKR1* axes NHIC/BPS: higher expression of *type I IFN* response genes, presence of *C37 + IGHD+* naïve B cells
Peng et al., 2022^15^	IC/BPS patients (n = 15); stress urinary incontinence patients (n = 9)	CD45+ cells from bladder tissue (n = 135.091)	IC/BPS: dysfunctional/exhausted CD8 + T cells (*R4A2, ISG20, TANK* and *CAPZB*), increased numbers of activated B cells and neutrophils, upregulated *CCL5*, decreased *IL10* and *IL35*, increased *TGFβ* in Tregs, upregulated receptors *CCR7*, *CXCR4, IFNGR1/2*, *TGFβR2* and *IL2RG*, infection with HPV2 in 95% IC/BPS patients
Peng et al., 2024^27^	HIC/BPS patients (n = 10), stress urinary incontinence patients (n = 7)	Urothelial cells from bladder tissue (n = 10.563)	HIC/BPS: reduced *UPK3A +* umbrella cells, increased progenitor‐like pluripotent cells (PPCs), *TLR3* and *NR2F6* associated with the injury of *UPK3A +* umbrella cells, downregulated *KRT20* and *ZO1*, upregulated *TNFα* and *IL6*.
Cheng et al, 2021^35^	Mouse models with acute (n = 4–6) and chronic (n = 4–6) CYP administration, healthy mice (n = 4–6)	Bladder tissue cells (n = 50.404)	CYP‐induced: cell cycle differences between basal and intermediate cells, basal cells express adhesion molecules *Itgb1, Itgav* and *Itgb5*, and undergo complete divisions compared with intermediate cells, which can directly differentiate into superficial cells through an incomplete cell cycle, basal cells undergo divisions for self‐renewal rather than differentiation, *Acta2 + Car3 +* myofibroblasts serve as a niche for proliferating urothelial cells by producing *Fgf7*, *Ugt2b34* was identified as a novel marker of intermediate cells.

IC/BPS, interstitial cystitis/bladder pain syndrome; NHIC, Non‐Hunner type; HIC, Hunner type; CYP, cyclophosphamide.

This review highlights the critical need for detailed mapping of cellular subsets, phenotypic changes, activation states, developmental pathways and intercellular communication to advance IC/BPS research. It categorizes scRNA‐seq studies by cell type to clarify the roles and interactions of various cell populations in healthy and IC/BPS‐affected bladders. Given the frequent use of mouse models to study IC/BPS mechanisms, we compare human and mouse bladder cell subtypes in disease conditions. In addition, we discuss sex‐based differences in bladder cell subtypes that may contribute to the higher prevalence of IC/BPS in females. Finally, we address future challenges and opportunities for large‐scale scRNA‐seq studies in this field.

## 2 SINGLE‐CELL LANDSCAPE OF BLADDER STRUCTURAL NON‐IMMUNE CELLS

### 2.1 Urothelial cells

The bladder urothelium is a stratified epithelium that forms a tight, impermeable barrier between blood and urine, preventing urine leakage into the underlying layers of the bladder.^22^ Based on anatomical location and the expression of a few marker genes, urothelial cells are traditionally classified into three groups: i) basal cells; ii) intermediate cells and iii) a luminal layer of fully differentiated superficial cells, known as umbrella cells.^23^ However, the exact number and characteristics of bladder urothelial cell subtypes remain unclear, requiring novel markers for their classification—a task now achievable with scRNA‐seq.

#### 2.1.1 Urothelial cell populations in patients with IC/BPS

Although bladder biopsies from human patients are challenging to obtain due to the invasive nature of the procedure, they remain essential for IC/BPS research. A predominant pathological feature of IC/BPS is urothelial denudation, characterized by thinning of the bladder urothelium and partial or complete loss of the superficial cell layer.^24^ Abnormal differentiation of the bladder urothelium has been observed in IC/BPS bladders, together with reduced expression of adhesion and tight junction proteins and uroplakins (UPK). This leads to compromised intercellular junction integrity, and along with a reduced urothelial cell population, impairs the urothelial barrier. As a result, solutes and toxic substances from urine infiltrate the urothelium and underlying layers, triggering inflammation.^25^, ^26^ Although various lines of evidence suggest that urothelial cells play a central role in the initiation and progression of IC/BPS, only one study to date has utilized scRNA‐seq to specifically examine urothelial cells in human HIC/BPS patients.^27^ This pioneering study identified eight distinct clusters of urothelial cells in the HIC/BPS‐affected bladders. A cluster of cells with high gene expression of *KRT5* and *KRT14*, markers associated with stem cells in the urothelium, along with *KRT13*, a marker of intermediate cells, was predominantly found in IC/BPS bladders. This cluster also expressed markers of proliferation, including *Ki‐67* (*MKI67*) and *proliferating cell nuclear antigen* (*PCNA*), as well as *grainyhead‐like transcription factor 3* (*GRHL3)*, which plays a crucial role in urothelial barrier formation. These cells were identified as progenitor‐like pluripotent cells (PPCs), distinguished by their strong stemness and proliferation capacity. Alongside an increase in PPCs, a significant reduction in *UPK3A +* umbrella cells was observed in IC/BPS bladders, suggesting that PPCs proliferated to compensate for the loss of *UPK3A +* cells. However, the damage to *UPK3A +* cells exceeded the proliferation rate of PPCs, leading to impaired urothelial regeneration and the onset of chronic inflammation. Key molecular pathways, including *Toll‐like receptor 3 (TLR3)* and *NR2F6*, were identified as playing a pivotal role in the injury to *UPK3A +* umbrella cells in HIC/BPS bladders, which was subsequently confirmed in experimental in vitro and in vivo models. This study proposes a model in which external factors, such as viral or endogenous ligands, activate TLR receptors expressed on umbrella cells, initiating downstream innate immune signalling. Supporting this, recent studies have identified high prevalence rates of EBV and HPV infections present in IC/BPS patients, particularly HIC/BPS, suggesting a possible viral aetiology in a subset of cases.^13^, ^15^ Moreover, Akiyama et al.^28^ recently reported upregulation of cyclic GMP‐AMP synthase (cGAS), the stimulator of interferon genes (STING) pathway and type I interferon (IFN) expression in HIC/BPS bladder tissue. These pathways can be activated by cytosolic DNA, including mitochondrial DNA (mtDNA), which may be released into the cytoplasm in response to cellular stress.^28^ Cell‐free mtDNA may serve as a damage‐associated molecular pattern (DAMP), recognized by TLRs and cytosolic sensors, thereby acting as a potent endogenous trigger of inflammation.^29^ Notably, oxidative stress, an established inducer of mtDNA release, has also been implicated in IC/BPS pathogenesis.^30^, ^31^

Activation of TLR3 signalling promotes NR2F6 transcription, which may modulate inflammatory gene expression. This cascade contributes to the disruption of the urothelial barrier and the secretion of pro‐inflammatory mediators from urothelial cells. Damage to UPK3A + umbrella cells, which are critical for barrier integrity, further compromises the urothelium, allowing urinary solutes to infiltrate subepithelial tissues and exacerbate inflammation. This establishes a positive feedback loop involving TLR3 signalling, NR2F6 activation and barrier dysfunction, potentially driving the chronic inflammatory state characteristic of HIC/BPS. Targeting the TLR3–NR2F6 axis may thus represent a promising therapeutic strategy to restore the urothelial barrier and alleviate lower urinary tract symptoms in IC/BPS patients.^27^ However, it is possible that PPCs have a more limited potency, potentially being unipotent or multipotent, which warrants further exploration in future studies.

#### 2.1.2 Urothelial cell populations in experimental mouse models

Experimental mouse models are commonly used to mimic an IC/BPS phenotype, typically through induced bladder injury by intravesical injections of cyclophosphamide (CYP).^32^ This model is among the most widely used and well‐characterized animal models for studying IC/BPS. Repeated low‐dose CYP injections over 2–3 weeks (mimicking chronic IC/BPS, observed in human patients) induce transient bladder inflammation, characterized by mild oedema, moderate upregulation of proinflammatory cytokines (e.g., IL1β, IL6, TNFα), mast cell infiltration and bladder overactivity. However, this model lacks key features of chronic IC/BPS, such as persistent urothelial damage, fibrosis and long‐term inflammation, as tissue regeneration typically occurs within 10 days post‐treatment. Thus, while useful for studying acute inflammatory responses, the CYP model does not fully replicate the chronicity and multifactorial nature of human IC/BPS. Alternative models attempt to address these limitations. Autoimmune‐based models more closely mimic chronic disease by activating both innate and adaptive immunity, leading to sustained urothelial barrier loss, fibrosis and persistent inflammation. Stress‐induced models simulate the impact of psychological and physical stressors, which are relevant particularly to NHIC/BPS. Each model captures distinct aspects of the disease, highlighting the need for a multifaceted preclinical approach. Ongoing debate persists regarding whether a single model can encapsulate IC/BPS heterogeneity or whether specific models are needed for different clinical subtypes and disease stages.^33^

Whereas in normal conditions, the urothelium remains quiescent, it has to regenerate rapidly in response to injury to maintain the blood‐urine barrier.^34^ While it was traditionally believed that KRT5‐expressing basal cells were the predominant cell type responsible for urothelial repair,^23^ Cheng et al.^35^ discovered that after CYP‐induced injury, two cycling cell populations belonging to basal and intermediate urothelial cells were significantly enriched in mice bladders. These two populations exhibited distinct division patterns, potentially linked to differences in cell adhesion. Several cell adhesion components, such as *Itgb1, Itgav* and *Itgb5*, were strongly expressed in cycling basal cells but not in intermediate cells, suggesting that basal cells undergo more complete divisions. By analysing the lineage relationships among urothelial cell subpopulations, the authors found that cycling intermediate cells can directly differentiate into superficial cells through an incomplete cell cycle, while cycling basal cells likely undergo divisions for self‐renewal rather than differentiation. Additionally, a novel marker of intermediate cells, *Ugt2b34*—associated with glycosylation—was identified, and it was not expressed in either basal or superficial cells.^35^

In healthy mouse bladders, Li et al.^36^ identified novel urothelial cell types with potential roles in bladder tissue immunity and regeneration. One cluster of cells expressed *Plxna4* in addition to *Upk3*, suggesting they belong to the superficial cell layer. Plxna4 is essential for optimal cytokine production upon TLR stimulation and bacterial challenge, indicating its crucial role in bladder tissue immunity. Another urothelial cell cluster was characterized by the marker gene *Aspm*, which encodes abnormal spindle microtubule assembly protein and may serve as a stem/progenitor cell marker. *Aspm+* urothelial cells were significantly increased in mouse bladders following acute uropathogenic *E. coli* infection, suggesting their involvement in urothelial regeneration. Furthermore, the study found that the expression of genes known to contribute to IC/BPS pathogenesis (e.g., *Anxa1*, *Tnfsf13b, Cdkn1a, Nqo1, Trpv4, Vegfa*) was significantly higher in basal cells compared to superficial urothelial cells. These results suggest that IC/BPS may originate in these subpopulations of bladder urothelium.^36^ However, given that IC/BPS is characterized by urothelial denudation, with superficial and intermediate cells reduced or absent,^24^ these findings likely reflect biopsies taken from denuded areas.

Although experimental mouse models are crucial for studying the pathobiology of IC/BPS, caution is necessary when translating these findings to human patients. Yu et al.^37^ found differences in the expression of certain keratins between quiescent human and mouse urothelial cells. Specifically, *KRT17* and *KRT13* were highly expressed in human urothelial cells but were expressed at lower levels in mouse urothelium, while *Krt15* expression was significantly higher in mouse urothelial cells compared to human epithelial cells. However, the expression levels of *KRT7, KRT8, KRT18, KRT19* and *KRT20* were similar in both human and mouse epithelial cells.^37^ These differences should be considered when using mouse models to study IC/BPS or other diseases affecting the human bladder.

### 2.2 Interstitial cells

The second layer of the bladder, located beneath the urothelium, is the lamina propria, a thin layer of connective tissue.^38^ The predominant cells in the lamina propria are fibroblasts, which are of mesenchymal origin. These fibroblasts can differentiate into other cell types, including myofibroblasts and smooth muscle cells, depending on the specific conditions in the tissue.^39^ In recent decades, the term interstitial cells has been used broadly to describe various mesenchymal‐derived cells within the bladder lamina propria. However, advances in single‐cell transcriptomics and imaging approaches now enable more precise classification of these cell types. Throughout this review, we refer to these cells as fibroblasts or myofibroblasts where specific molecular identities have been established.

It is important to note that the lamina propria also contains immune cells (e.g., mast cells, macrophages) and vascular elements, which may not be of mesenchymal origin. Together, these cells perform diverse and multifunctional roles, including participation in tissue remodelling, fibrosis, repair processes and signalling interactions with urothelial and smooth muscle cells.^38^

Although interstitial cells have been the subject of extensive research, their precise roles, localization and phenotypic identities remain incompletely understood.^40^, ^41^, ^42^ Clarifying their specific locations, molecular profiles and functional properties in both health and disease is essential for advancing our understanding of bladder pathophysiology.

#### 2.2.1 Interstitial cell populations in patients with IC/BPS and experimental mouse models

Using scRNA‐seq and imaging mass cytometry, Su et al.^43^ identified three distinct subtypes of fibroblasts in the bladders of female patients with IC/BPS. In line with this, we refer to these subtypes explicitly as fibroblasts rather than using the broader term interstitial cells, as their identity was supported by transcriptomic and proteomic markers. These subtypes include: i) *PDGFRA+* fibroblasts, which exhibit high expression of key collagen genes, including *COL1A1*, *COL1A2 and COL3A1*; ii) *RGS5 +* fibroblasts, which display features similar to vascular smooth muscle cells, with notable expression of *ACTA2*, *TAGLN* and *MYL11*; and iii) pro‐inflammatory fibroblasts, characterized by strong expression of chemokine genes such as *CCL2, CXCL2* and *CXCL8*.^43^ Contrary to previous reports identifying macrophages, lymphocytes or mast cells as the main sources of IL6 in IC/BPS rat models,^44^, ^45^ Su et al.^42^ demonstrated that pro‐inflammatory fibroblasts are the predominant *IL6* producers. These fibroblasts, which are in an active cytokine‐secreting state resembling cancer‐associated fibroblasts, may represent a novel therapeutic target for IC/BPS.^43^

Zhao et al.^46^ investigated the differences between CYP‐induced acute and chronic bladder injury in mice and identified two distinct types of interstitial cells. To distinguish between cell types within the interstitial compartment, Zhao et al. used single‐cell profiling to clearly define fibroblasts and myofibroblasts based on distinct marker expression. We therefore adopt this specific nomenclature when referring to these populations and reserve the term interstitial cells only for contexts where further subclassification is not available. Fibroblasts were characterized by the markers *PiI16, Pdgfrα and Cd34*, while myofibroblasts were identified by *Cd362* and *Cd121a*. In humans, myofibroblasts were found to specifically express transient receptor potential ankyrin 1 (*TRPA1*), which is involved in sensory and inflammatory signalling. However, the expression of *Trpa1* was undetectable in myofibroblasts from mouse bladder tissue. Interestingly, myofibroblasts were predominantly present in mice with acute CYP‐induced injury but diminished in chronic CYP injury.^46^ Myofibroblasts are contractile, extracellular matrix‐secreting cells that can arise from various precursors, including fibroblasts, and smooth muscle cells. Their presence is critical during acute injury, facilitating tissue remodelling and repair. However, persistent activation of myofibroblasts can contribute to fibrosis under chronic inflammatory conditions.^47^ The diminished presence of myofibroblasts in the bladders of mice with chronic CYP‐induced injury suggests they may not play a significant role in the pathophysiology of IC/BPS. However, as these findings were derived from mouse models, the species‐specific differences in the roles and functions of myofibroblasts highlight the need for further investigation into the translational relevance of mouse models for understanding human bladder disorders.

## 3 SINGLE‐CELL LANDSCAPE OF BLADDER IMMUNE CELLS

Despite the high prevalence of bladder infections, comprehensive data profiling of bladder‐resident immune cells in both homeostasis and disease remains limited. This highlights the evolutionary drive on urothelium to be regenerative and to limit inflammation, as reflected in the scarcity of resident immune cells in healthy human urothelium. However, when the urothelial barrier fails, urine infiltrates underlying tissues, triggering inflammation and symptoms of cystitis.^48^ Earlier studies analysed single‐cell suspensions from mouse bladders using flow cytometry and identified a restricted range (macrophages, dendritic cells, T cells, B cells, NK cells) of tissue‐resident immune cells.^49^ A more recent scRNA‐seq study of sorted CD45 + cells from mouse bladders revealed a significantly more diverse immune landscape than previously recognized, also revealing previously unreported subsets of macrophages, dendritic cells and group 2 innate lymphoid cells.^50^

### 3.1 Immune cell populations in patients with IC/BPS

A detailed characterization of immune cell subtypes in IC/BPS came in 2022, when Su et al.^43^ provided a high‐resolution, unbiased mapping of immune cell profiles in five treatment‐naïve females with HIC/BPS, as well as NHIC/BPS. This study showed that Th1 cells, a subtype of CD4 + T cells, were strongly activated and predominantly located in the urothelial region of IC/BPS‐affected bladders. A Th1‐biased immune response was further indicated by the elevated expression of transcription factors *TBX21* and *RUNX3*, which drive the conversion of regulatory T cells (Tregs) into Th1 cells. Conversely, the number of Tregs with immunosuppressive functions was significantly reduced.^43^

Tissue‐resident macrophage‐associated genes were enriched in processes such as granulocyte chemotaxis, antigen processing and neutrophil degranulation, indicating their pivotal role in IC/BPS pathogenesis. These macrophages in IC/BPS patients expressed high levels of pro‐inflammatory cytokines, such as *TNFα* and *IL1β*, while pro‐inflammatory fibroblasts expressed elevated levels of *IL6*.^43^ Notably, Peng et al.^27^ reported upregulation of both TNFα and IL6 in urothelial cells from IC/BPS bladders, highlighting a pronounced pro‐inflammatory response in the urothelial compartment as well.^27^ While elevated levels of IL1β, IL6 and TNFα have previously been detected in the urine and serum of IC/BPS patients,^51^, ^52^ the specific cellular sources of these cytokines have only been identified recently in scRNA‐seq studies. Moreover, Tian et al.^53^ identified IL6 as one of the most effective urinary biomarkers for distinguishing HIC from other IC/BPS subtypes. This could reflect underlying pro‐inflammatory fibroblast activity, particularly in HIC/BPS, as revealed by the scRNA‐seq analysis. In addition, urinary IL6 could be used as a marker of treatment response to injections with platelet‐rich plasma (PRP) since its concentrations increased in non‐responders after PRP injection.^54^ Taken together, these findings suggest that the bladders of HIC/BPS patients are likely enriched in pro‐inflammatory fibroblasts, contributing to both inflammation and fibrosis. Consequently, this subgroup of patients may benefit more from IL6‐targeted therapies than other IC/BPS subtypes. Furthermore, this distinct fibroblast population may drive disease progression by amplifying the inflammatory response and could serve as a prognostic marker for disease severity or therapeutic response in HIC/BPS patients.

Although TNFα signalling has long been recognized as significant in IC/BPS, Su et al.^43^ suggested that blocking TNFα alone is insufficient to address the complex pathogenic cellular organization of the bladder. This is supported by findings from a phase III, randomized, double‐blind, placebo‐controlled trial in which adalimumab (a monoclonal antibody targeting TNFα) did not show statistically significant improvement in any outcome measure compared to placebo.^55^ A combined therapeutic approach targeting TNFα, IL1β and IL6 may be more effective in treating IC/BPS.^43^ For example, phosphodiesterase four inhibitors, which simultaneously block TNFα and IL6 could be used for the treatment of IC/BPS. Some of them (oflumilast, apremilast and crisaborole) are already approved for the treatment of inflammatory airway diseases, psoriatic arthritis and atopic dermatitis.^56^

The same study identified an inflammatory component in NHIC/BPS patients without Hunner lesions, challenging the previous belief that this subtype lacks bladder inflammation. While Maeda et al.^24^ found no signs of inflammation in 39 NHIC/BPS bladder biopsies, Su et al.^43^ observed that *C37 +* naïve B cells, expressing the *immunoglobulin heavy constant delta (IGHD)* gene, were the predominant B‐cell subset in NHIC/BPS biopsies but were absent in HIC/BPS samples. Additionally, genes in the IRF family (*IRF2, IRF4 and IRF8)*, key transcriptional regulators of interferons (IFNs) and IFN‐inducible genes, were identified as specific regulons associated with NHIC/BPS. These genes play a crucial role in T‐cell differentiation during inflammation and suggest a type I IFN response in NHIC/BPS.^43^ Previously, there was debate over whether NHIC/BPS should be considered a milder form of HIC/BPS or a distinct pathological entity. However, these findings indicate that NHIC/BPS also exhibits an inflammatory component, which may have been overlooked due to the limitations of standard, less sensitive techniques.

By integrating scRNA‐seq, spatial transcriptomics and cytometry by time of flight (CyTOF), Peng et al.^15^ identified high numbers of dysfunctional/exhausted CD8 + T cells in bladder biopsies from 15 female IC/BPS patients. Exhausted CD8 + T cells in IC/BPS expressed activation‐related genes such as *TRAC*, *TRBC1* and *ACTB*, yet displayed low cytotoxic activity and elevated levels of exhaustion markers, including *NR4A2, ISG20, TANK* and *CAPZB*. These cells, characterized by diminished effector function due to chronic antigenic stimulation, are well‐documented in infections, cancer and autoimmune or chronic inflammatory conditions.^57^, ^58^ This exhausted phenotype, observed for the first time in IC/BPS, could serve as a molecular signature for identifying patients with chronic immune activation. Importantly, CD8 + T cell exhaustion is a potentially reversible state, particularly through therapeutic blockade of persistently expressed inhibitory receptors such as PD‐1. Stratifying IC/BPS patients based on the prevalence of exhausted CD8 + T cells could facilitate the design of targeted clinical trials and identify those most likely to benefit from immunotherapeutic interventions.

Consistent with previous findings,^43^ Tregs in IC/BPS bladders demonstrated impaired function, as evidenced by decreased expression of *IL10* and *IL35*, alongside upregulation of the *TGFβ* signalling pathway. This highlights the broader dysregulation of immune regulatory mechanisms contributing to IC/BPS pathogenesis.^15^

Most of the myeloid cells in IC bladders were neutrophils (51%) instead of mast cells which were previously reported as the dominant immune cell population in IC/BPS bladders.^59^ Nearly all immune cell populations in IC/BPS bladders had upregulated gene expression of *CCL5* and several other chemokine/cytokine receptors (*CCR7, CXCR4, IFNGR1/2, TGFβR2* and *IL2RG*), indicating they could serve as potential therapeutic targets. Increased protein levels of CCL5 were also noted in the urine sample of IC/BPS patients, confirming previous studies.^31^, ^51^

**FIGURE 1:**
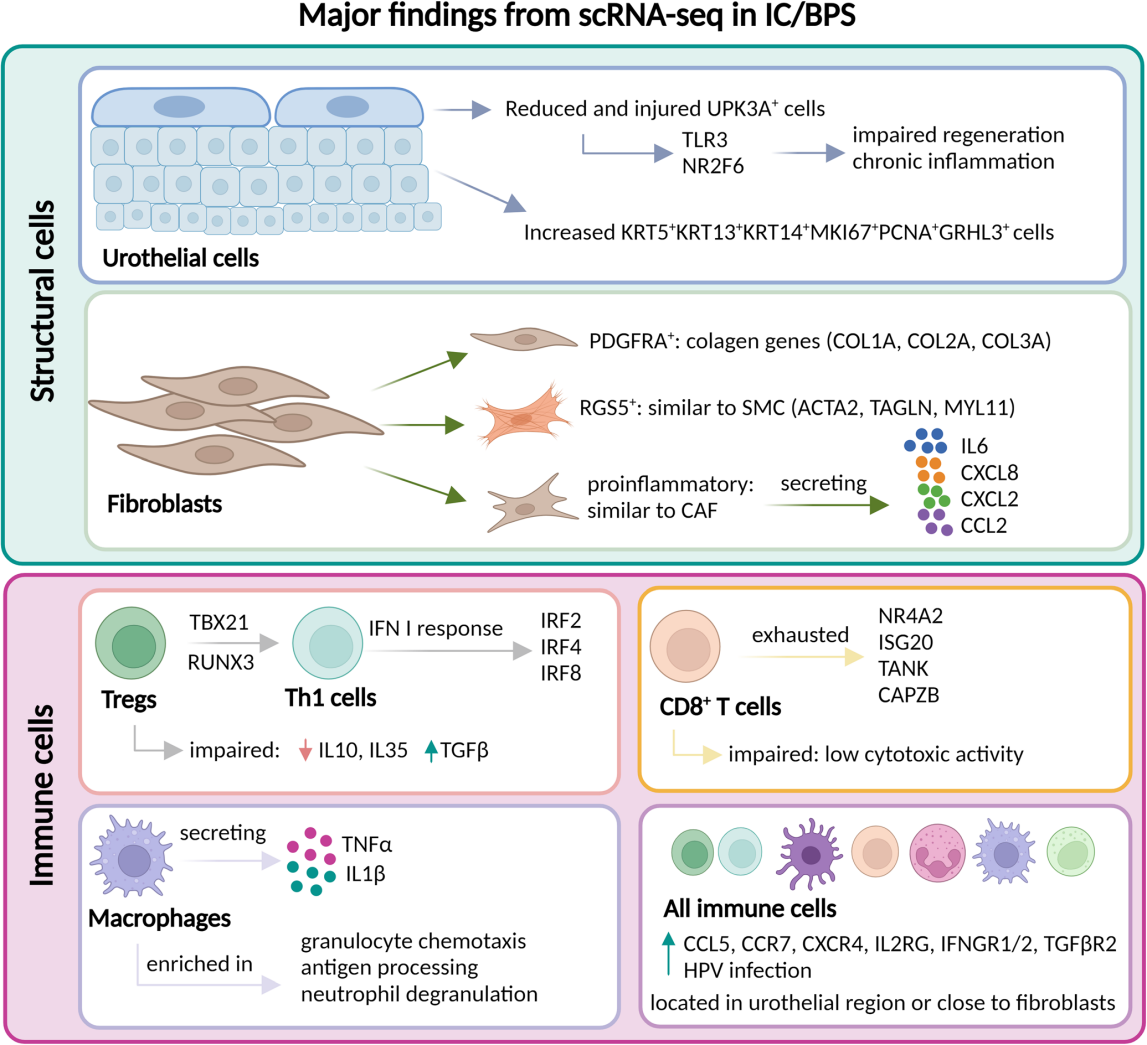
**Summary of the major findings resulting from scRNA‐seq application in patients with IC/BPS.** ScRNA‐seq can be used to characterize the heterogeneity and function of structural and immune cells, identify rare cells and new cell subtypes, as well as explore the communication between cells. This figure was created using Biorender.com. IC/BPS, interstitial cystitis/bladder pain syndrome; scRNA‐seq, single‐cell RNA sequencing.

Enrichment analysis revealed that the differentially expressed genes between IC/BPS and controls across various immune cell populations were associated with HPV infection. HPV DNA was subsequently detected in 19 out of 20 urine samples from IC/BPS patients but in none of controls, offering novel evidence for a potential viral origin of IC/BPS.^15^ Major findings from scRNA‐seq data in IC/BPS patients are summarized in Figure 1.

The majority of immune subpopulations identified were enriched in the urothelial region or located near fibroblasts in IC/BPS bladders. In contrast, only half of the immune cell subsets were present in the urothelial region of control bladders, suggesting that fibroblasts and urothelial cells play a crucial role in shaping the bladder tissue immune response in IC/BPS. While this study highlights the significant role of inflammation and immunity in IC/BPS progression, it also suggests that these processes are likely downstream effects of epithelial barrier destruction.^15^ Identifying the underlying causes of urothelial injury is therefore essential for the development of more effective therapies in IC/BPS.

### 3.2 Immune cell populations in experimental mouse models

To date, no study using scRNA‐seq has specifically focused on the immune cell landscape in mouse models of IC/BPS. However, given that IC/BPS most commonly occurs in older women, potentially linked to age‐associated disruptions in the immune response, studying the immune microenvironment in young versus aged mice at single‐cell resolution could provide valuable insights into the origin and development of IC/BPS.^60^, ^61^ Ligon et al.^50^ first reported that aged mice (18 months) exhibit expanded B‐ and T‐cells that form structures called bladder tertiary lymphoid tissue (bTLT). These bTLTs in aged mice act as centres for B‐cell recruitment, activation and differentiation into plasma cells. A small group of macrophages expressing *Cxcl13*, a chemokine involved in lymphocyte organization, was found exclusively in the bladders of aged mice, suggesting these macrophages play a key role in bTLT formation. This discovery marks a significant shift in the immune landscape of the ageing bladder, as organized lymphoid tissue is normally absent in healthy bladders. TNFα, a key inflammatory mediator linked to bTLT formation and ageing, was identified in this process.^50^ TNFα is also a major contributor to the inflammatory response in IC/BPS^19^; however, bTLT‐like structures have not yet been reported in the bladders of IC/BPS patients.

A recent study confirmed an expanded number of T‐ and B‐cells in the bladders of aged mice and identified the chemokine receptor *Ccr7* as a key mediator of T‐ and B‐cell infiltration.^61^ Higher expression of *CCR7* and its ligand *CCL19*, both associated with T‐ and B‐cell infiltration was also found in the bladders of IC/BPS patients.^61^, ^62^ Molecular docking experiments identified triptolide as a promising therapeutic agent, as it can bind to both CCR7 and CCL19.^62^ Altogether, these findings highlight significant changes in the bladder mucosal immune system of aged mice, potentially linking aging to IC/BPS.

## 4 CELL‐TO‐CELL COMMUNICATION IN THE BLADDER

In 2022, Shi et al.^63^ compiled an extensive dataset comprising over 23 000 mouse cells and 29 000 human cells derived from 11 bladder samples across various datasets. The authors created the largest integrated bladder dataset to date, encompassing urothelial, interstitial and immune cells, along with a comprehensive analysis of cell‐to‐cell interactions. Significant interactions were identified between basal urothelial cells and smooth muscle cells in human bladders, with bi‐directional communication involving genes such as *thrombospondin (THBS), midkine (MK), fibronectin 1 (FN1)*, NAMPT encoding *visfatin* and *laminin (LAM)*, the latter being the most prevalent.^63^

In mouse bladders, enhanced communication was observed between basal urothelial cells and fibroblasts through the non‐canonical Wnt (ncWnt) pathway, *macrophage migration inhibitory factor*, Nectin pathways and several extracellular matrix (ECM) receptors, including THBSLAM, *collagen, FN1, tenascin, MK* and *galectin*. Among the enriched ligand‐receptor pairs identified, 24.7% (66 out of 268) of receptors in mice and 23.9% (102 out of 426) in humans were directly linked to the integrin superfamily. This finding underscores the critical role of integrins in cell communication networks within the bladder.^63^

Previous studies have shown that enhanced communication between human bladder smooth muscle cells and myofibroblasts via gap junctions is directly linked to IC/BPS and overactive bladder syndrome. This communication can be modulated by various cytokines; for instance, IL6 promotes cell‐to‐cell communication, while IL4 and TGFβ inhibit it.^64^ A key difference in cellular communication in the bladders of IC/BPS patients compared to controls was described by Su et al.^43^ In IC/BPS, there is enhanced communication between macrophages and endothelial cells, whereas in controls, structural cells (e.g., fibroblasts, endothelial cells and urothelial cells) primarily drive cell interactions. The study identifies the *CXCL8‐ACKR1, CXCL2‐ACKR1* and *CXCL3‐ACKR1* ligand‐receptor pairs as critical contributors to macrophage‐to‐endothelial cell signalling in IC/BPS patients.^43^ Importantly, CXCL8, CXCL2 and CXCL3 are potent chemoattractants for neutrophils, capable of inducing degranulation and morphological changes and facilitate their transendothelial migration. Since macrophages are among the first responders to antigenic stimuli, they are likely the initial source of CXCL8 in the bladder microenvironment.^65^ This is consistent with findings from inflamed intestinal mucosa, where vessel‐associated macrophages prime endothelial cells for neutrophil TEM by inducing ICAM‐1 hot spots, a process dependent on macrophage‐derived TNFα and endothelial TNFR2.^66^ Given the increased neutrophil infiltration observed in IC/BPS,^15^ this macrophage–endothelial axis likely plays a central role in neutrophil recruitment and sustained inflammation.

## 5 SEX DIFFERENCES IN BLADDER CELL TYPES

Some bladder‐related diseases, including IC/BPS have significant sex differences in incidence being more prevalent in females.^67^ However, the molecular mechanisms underlying these differences remain poorly understood. A recent paper by Wu et al.^68^ addressed this issue by analysing scRNA‐seq data from normal human bladders of three females and three males. Differential gene expression between sexes was predominantly observed in urothelial cells, fibroblasts, B‐cells and T‐cells. In female urothelial cells, genes associated with bacterial response and intrinsic apoptotic signalling were upregulated. Conversely, male urothelial cells showed significant upregulation of genes and pathways related to ribosome biogenesis, a process closely linked to cell growth and proliferation. Indeed, a greater proportion of proliferating urothelial cells was identified in males (55%) compared to females (35%), suggesting enhanced cellular self‐renewal and regeneration in male bladders after injury. In contrast, female bladders showed reduced proliferative activity and impaired regenerative capacity, which may compromise urothelial barrier integrity.^68^ Supporting this, a marked reduction in UPK3A^+^ urothelial cells was observed in IC/BPS bladders by Peng et al.^27^ The loss of UPK3A expression reflects disrupted urothelial differentiation and structural integrity, further weakening the barrier and facilitating chronic inflammation. Together, these findings might provide a mechanistic explanation for the higher prevalence of IC/BPS in females: the combination of diminished regenerative potential, reduced UPK3A^+^ cell numbers and compromised barrier function may underlie their increased susceptibility to injury. In contrast, the enhanced ribosome‐driven regeneration in males likely supports more efficient urothelial repair and maintenance of barrier integrity, offering greater resilience against disease onset and progression. Compared to males, females exhibited higher numbers of activated T‐cells, B‐cells and plasma cells, enabling a more robust immune response to external stimuli.^68^ This adaptive immune activation alongside a shift in cellular communication networks toward enhanced signalling between macrophages and endothelial cells^15^, ^43^ also occurs in IC/BPS bladders. Activated macrophages and endothelial cells may promote vascular permeability, and immune cell recruitment, thereby setting the stage for the infiltration and activation of T‐cells, B‐cells and plasma cells. Thus, the increased presence of activated adaptive immune cells in females and the macrophage‐endothelial signalling axis in IC/BPS are likely functionally linked, collectively contributing to chronic inflammation, urothelial barrier disruption and disease progression.

Similar findings were reported in a mouse model of CYP‐induced IC/BPS, where urothelial proliferation was more pronounced in male bladders, while innate immunity and tissue remodelling processes were enriched in females.^69^ Furthermore, healthy female bladders displayed a less robust barrier function compared to males, as evidenced by lower transepithelial electrical resistance (TEER). TEER measurements also revealed delayed barrier recovery in chronically inflamed female bladders compared to males.^70^ While these studies provide valuable insights into sex‐specific mechanisms underlying IC/BPS pathology, future research should also consider age as a crucial factor. Incorporating both sex and age into the analysis could provide a more comprehensive understanding of the disease, leading to even more targeted and effective therapeutic strategies.

## 6 LIMITATIONS OF USING sc‐RNA‐seq

Despite its promise, the routine clinical adoption of scRNA‐seq still faces substantial financial, technical and logistical barriers. These include high costs, limited scalability and the need for standardized protocols and user‐friendly analytic tools that can be integrated into clinical workflows. Additionally, scRNA‐seq has inherent biological and technical limitations. The loss of spatial context due to tissue dissociation can obscure the native cellular organization and microenvironmental interactions. The tissue dissociation itself can introduce bias, as certain cell types, especially terminally differentiated umbrella cells, may be underrepresented due to their susceptibility to enzymatic or mechanical disruption. However, these limitations can be prevented by incorporation of spatial transcriptomics or alternatively, using single‐nucleus RNA‐seq protocols, which can be applied to snap‐frozen samples, thus avoiding many of the dissociation‐related artefacts.^57^ Technical constraints such as dropout events, where mRNAs present in a cell are not detected due to low abundance or inefficient capture, can lead to false negatives. In addition, most high‐throughput scRNA‐seq platforms sequence only a fragment of each transcript, limiting the ability to assess alternative splicing, isoform usage or RNA editing. Biologically, scRNA‐seq provides only a static snapshot of gene expression, making it difficult to capture dynamic cellular transitions over time, such as inflammation, differentiation or repair processes. While pseudotemporal algorithms attempt to predict lineage trajectories, these remain only approximations without direct validation.^58^

## 7 CONCLUSIONS AND AN OUTLOOK FOR THE FUTURE

Recent advances in transcriptomics have shifted from bulk population studies to scRNA‐seq, revolutionizing our understanding of biological systems. Despite its brief history, scRNA‐seq has driven groundbreaking discoveries, enabling the identification of novel cell populations, lineage trajectories and intercellular communication across developmental and disease contexts. In the field of bladder diseases, scRNA‐seq has been pivotal in uncovering novel cell types and producing single‐cell atlases, now widely accessible for reference and further research. Although technical challenges remain, improvements in experimental and analytical pipelines are expanding the scope of scRNA‐seq. Integrating scRNA‐seq with other single‐cell techniques (e.g., genomics, scATAC‐seq) promises to elucidate gene regulatory mechanisms and enhance precision medicine. Combining these tools with bulk RNA‐seq, spatial transcriptomic, imaging and functional studies will deepen insights into mechanisms underlying IC/BPS, and aid therapeutic development.

A significant challenge in bladder research is the difficulty of obtaining tissue samples from both patients and healthy individuals, limiting the availability of high‐quality material for scRNA‐seq studies. This highlights the potential of urine‐derived scRNA‐seq as a non‐invasive and high‐resolution alternative for studying bladder biology and pathology. Recent studies have already demonstrated the feasibility of this approach in addressing kidney disease^71^ and bladder cancer,^72^ paving the way for broader applications in other bladder‐related conditions. Urine‐derived single‐cell data could expand the accessibility of this technology to a larger patient cohort. This will not only enhance our understanding of IC/BPS pathogenesis, but also significantly facilitate its diagnosis. Furthermore, as our understanding of the disease at the molecular level deepens, we may be able to refine the definition of IC/BPS, which currently encompasses multiple subtypes within a broad clinical spectrum. This could enable more personalised therapeutic approaches and improved clinical trial designs, ultimately leading to more effective treatments.

## AUTHOR CONTRIBUTIONS

**Tadeja Kuret:** Conceptualization; methodology; investigation; writing—original draft; writing—review and editing; visualization; project administration. **Mateja Erdani Kreft:** Conceptualization; writing—review and editing; project administration.

## CONFLICT OF INTEREST STATEMENT

The authors declare no conflict of interest.
